# Rate of conversion and complications of laparoscopic cholecystectomy in a tertiary care center in Saudi Arabia

**DOI:** 10.4103/0256-4947.60521

**Published:** 2010

**Authors:** Wagih Ghnnam, Jawid Malek, Emad Shebl, Turky Elbeshry, Ahmad Ibrahim

**Affiliations:** a From the Department of General Surgery, Mansoura Faculty of Medicine, Mansoura, Egypt; b From the Department of General Surgery, Khamis General Hospital, Khamis Mushayt, Saudi Arabia

## Abstract

**BACKGROUND AND OBJECTIVES:**

Problems during laparoscopic cholecystectomy include bile duct injury, conversion to open operation, and other postoperative complications. We retrospectively evaluated the causes for conversion and the rate of conversion from laparoscopic to open cholecystectomy and assessed the postoperative complications.

**METHODS:**

Of 340 patients who presented with symptomatic gall bladder disease over a 2-year period, 290 (85%) patients were evaluated on an elective basis and scheduled for surgery, while the remaining 50 (14.7%) patients were admitted emergently with a diagnosis of acute cholecystitis.

**RESULTS:**

The mean age of the patients was 41.9 (12.6) years. Conversion to laparotomy occurred in 17 patients (5%). The incidence of complications was 3.2%. The most common complication was postoperative transient pyrexia, which was seen in four patients (1.2%) followed by postoperative wound infection in three patients (0.9%), postoperative fluid collection and bile duct injury in two patients each (0.6%).

**CONCLUSION:**

Laparoscopic cholecystectomy remains the ‘gold standard’ by which all other treatment modalities are judged. Conversion from laparoscopic to open cholecystectomy should be based on the sound clinical judgment of the surgeon and not be due to a lack of individual expertise.

Laparoscopic cholecystectomy (LC) is one of the most commonly performed laparoscopic procedures. It requires only a small wound (0.5-1 cm), causes relatively less pain, allows for early ambulation, requires shorter hospital stay and therefore allows early return to work, and is associated with an early return of intestinal movement and a lower incidence of incisional hernia.^1^–^3^ The complications encountered during LC are numerous: some that are specific to this unique technique and some that are common to laparoscopic surgery in general. These include complications related to anesthesia; complications related to peritoneal access (e.g., vascular injuries, visceral injuries, and port-site hernia formation); complications related to pneumoperitoneum (e.g., cardiac complication, pulmonary complications, and gas embolism); and complications related to thermocoagulation. Specific complications of LC are hemorrhage, gall bladder perforation, bile leakage, bile duct injury, and perihepatic collection), and others such as external biliary fistula, wound sepsis, hematoma, foreign body inclusions, adhesions, metastatic port-site deposits, and cholelithoptysis.^4^,^6^ We evaluated the causes for conversion and the rate of conversion from laparoscopic to open cholecystectomy and describe the diagnosis and management of the complications of laparoscopic cholecystectomy.

## METHODS

We retrospectively reviewed 340 patients with symptomatic gall bladder disease who underwent LC at Khamis General Hospital between May 2006 and June 2008. Informed consent was obtained from all patients after the nature of the procedure and the possibility of the need for conversion from the laparoscopic approach to an open cholecystectomy was explained. A routine history was taken from all patients presenting for treatment of symptomatic gallbladder disease and all underwent a physical examination, laboratory testing, and ultrasonographic examination of the abdomen. Two hundred ninety (85%) patients were evaluated on an elective basis and scheduled for surgery, while the remaining 50 (14.7%) patients were admitted emergently with a diagnosis of acute cholecystitis. All acute cholecystitis patients (50) had right upper quadrant pain with localized tenderness. Twenty patients (40%) had fever of 38°C or greater on admission. An elevated white blood cell count (>10 000/mL) was present in 27 patients (54%). Ultrasonography showed wall thickening, pericholecystic fluid, and positive sonographic Murphy sign in 28 patients (56%). Patients admitted with a diagnosis of acute cholecystitis were treated with intravenous fluids, systemic antibiotics, and urgent surgery within 72 h of presentation. The clinical diagnosis of acute cholecystitis was confirmed by gross examination and histology in all 50 patients. All patients had surgery performed under general anesthesia. All were prepared and draped as for routine open cholecystectomy. A nasogastric (or orogastric) tube was inserted in those patients in whom there was a dilated stomach. An antibiotic (1 g Cephradine IV) was administered preoperatively, and a four-port technique was used in all the 340 patients.^7^,^8^ Pneumoperitoneum, using carbon dioxide, was established using standard laparoscopic techniques through either a ‘closed’ or ‘open’ approach. Pneumoperitoneum was established by a closed technique in 210 patients (61.8%), while an open approach was used in 130 patients (38.2%).

Data are presented as the mean (and standard deviation) or percentage. Statistical analysis was performed using SPSS version 17 (Statistical Package for Social Sciences, Chicago, IL, USA). *T* test was used to compare continuous variables and nonparametric tests were used where applicable.

## RESULTS

Of the 340 patients, 314 were women (92.4%) with a mean (SD) age of 41.4 (12.5) years and 26 were men (7.6%) with a mean age of 48.2 (13.19) years. The female-to-male ratio was 12:1. Indications for surgery were chronic calcular cholecystitis in the 290 patients (85.0%) who presented on an elective basis; the remaining 50 patients (14.7%) were admitted for treatment of acute cholecystitis. Ultrasonography demonstrated cholelithiasis in all the cases.

Conversion to laparotomy occured in 17 patients (5%) (Table 1). Perioperative complications leading to immediate or delayed laparotomy included injury to the common bile duct (two patients) and bile leakage due to a loose clip on the cystic duct in one patient. The incidence of complications was 3.2%. The most common complication was postoperative transient pyrexia, which was seen in four patients (1.2%) (Table 2).

**Table 1:** Indications for conversion from laparoscopic cholecystectomy to open cholecystectomy.

Factors contributing to conversion	Number of patients (%) (n = 340)
Contracted very small fibrotic adherent gall bladder	3 (0.9)
Gallbladder empyema	3 (0.9)
Unclear anatomy (acute cholecystitis)	6 (1. 7)
Difficult dissection	3 (0.9)
Gallbladder mass (cancer)	1 (0.3)
Common bile duct injury	1 (0.3)

**Table 2:** Postoperative complications.

Complication	Number of patients (%) (n=340)
Retained stones in common bile duct	2 (0.6)
Common bile duct injury	2 (0.6)
Biliary collection	1 (0.3)
Wound infection	3 (0.9)
Postoperative pyrexia	4 (1.2)

**Table 3:** Characteristics of laparoscopic and open cholecystectomy groups.

	Laparoscopic (n=323)	Converted (n=17)*	*P*
Hospital stay	1.4 (0.2)	5.9 (3.0)	.003
Mean age	41.7 (12.5)	47.2 (14.8)	.001
Operative time	43.0 (9.7)	118.0 (26.3)	.001
Males (n=26)	14	12	.007
Females (n=314)	309	5	

Values are mean (standard deviation) or number of patients.

* Ten cases were emergent (20% of e mergency cases) and seven were elective (2.4% of elective cases).

Among the 17 patients who underwent conversion to laparotomy, one patient developed wound infection. Two patients who underwent LC developed port-site infection and were treated conservatively. The mean hospital stay was less in the LC group than in the ‘converted to laparotomy’ group (Table 3). Of the two cases of bile duct injury, one case was diagnosed intraoperatively and was converted to open laparotomy with repair of the duct and T-tube insertion until healing; the T-tube was removed 10 days later, with no morbidity. The other case was diagnosed postoperatively, the patient presenting with jaundice, abdominal pain, and distension. This patient made a good recovery after laparoscopic exploration, with intrabdominal drain insertion, clipping of the leaking duct opening, and referral to a higher center for hepaticojejunostomy. Twenty percent of the emergent laparoscopic cholecystectomies were converted to open cholecystectomy, while only 2.4% of elective LCs were converted to open laparotomy. The conversion rate was more in males (12/26, 46%) than in females (5/314, 1.6%) (Table 3).

## DISCUSSION

Over the last two decades, LC has gained worldwide acceptance as the ‘gold standard’ in the surgical management of symptomatic cholecystolithiasis.^9^ It is the commonest operation performed laparoscopically worldwide.^10^ The conversion rate and complications associated with LC depend on the experience of the surgeon and the degree of difficulty faced during surgery. Different centers have reported widely varying rates of conversion to open operation (range: 1.5% to 6%).^11^,^12^

In our series, patient age was a significant predicting factor for difficulty during surgery and conversion to open cholecystectomy, with older patients being at greater risk for conversion to open cholecystectomy. Liu et al.,^13^ Simopolous et al.,^14^ and Kanaan et al.^15^ reported that patients treated successfully by LC were generally younger than 50-60 years of age; in comparison, patients who required conversion had a mean age of more than 50 years and had a history of recurrent attacks of cholecystitis. Wiebke et al.^16^ and Rosen et al.^17^ reported that male sex was not a risk factor for difficulty during surgery or conversion. On the other hand, the findings of Kanaan et al.,^15^ Simopolous et al.,^14^ and Nachnani and Supe^18^ are in agreement with our finding that male patients have an increased risk of difficult LC and that being a male increased the risk of unsuccessful LC.

As expected, an intraoperative finding of a chronically inflamed contracted gallbladder with a thickened wall during LC was associated with an increased conversion rate. In these cases, the dense adhesions signifed chronic inflammation and were certainly due to the repeated attacks of acute cholecystitis. Rosen et al.^17^ reported that conversion from laparoscopic to open cholecystectomy was required in 5.3% of their patients. The main indication for conversion in their series was severe inflammation, preventing accurate delineation of ductal anatomy. Kuldip and Ashish^19^ reported that the overall conversion rate was 0.4% of the total LCs performed and 1.7% of the difficult cases. They reported that dense adhesions in the region of the triangle of Calot was the leading cause for conversion to open surgery in 16.7% of their cases. Takegami et al.^20^ reported a conversion rate of 13% in LC performed by general surgeons and 2% in LC performed by specialized surgeons, suggesting that the skill of the operator has a large influence on the conversion rate. Meshikhes et al.^21^ and Al-Saigh et al.^22^ from Saudi Arabia reported a conversion rate of 11% in their cases, the most common cause of conversion being difficult anatomy. Conversion from LC to open cholecystectomy was required in 5% of our patients. This compares favorably with the rates reported in the literature.

Since surgeons are reluctant to publish their own rate of complications, and since the complications of LC are treated in tertiary care centers, the precise magnitude of the problem remains uncertain. Serious complications of LC occur in fewer than 2% of all cases.^23^ In our study the complication rate (3.24%) was within the range of published reports (0-8.6%), primarily because we included all complications, even the minor ones that were not related to the laparoscopic procedure itself. Placement of the Veress needle and the first trocar is usually linked to a complication rate that ranges from 0.3% to 0.5%.^24^ A survey performed in the US showed a risk of vascular injury of 0.5%, while the rate of visceral injury was between 0% and 0.4%.^25^ The reported incidence of bile duct injury is between 0% to 1% in LC.^22^,^23^,^26^,^27^ In our study the incidence was 0.6%.

Wound infection, which usually involves the cannulation port and the epigastric port through which the gallbladder is extracted, occurs in 0.3% to 1%^27^ of cases. In our study it occurred in three cases (0.9%): two in the port-site and one in the converted group. All were treated successfully with intravenous antibiotics. Postoperative fever occurred in 2.5% of patients in one study.^27^ In our study it occurred in 1.8% of cases, and in all cases it was associated with either wound infection or immediate postoperative fluid collections. Other complications, such as those related to pneumoperitoneum or thermocoagulation, were not seen in our study. Port-site hernia was not found in our study, possibly because of the lack of long-term follow-up.

Conversion from LC to open cholecystectomy should be based on the sound clinical judgment of the operating surgeon and not be because of lack of expertise.
